# General practitioners' evaluation of community psychiatric services: responsiveness to change of the General Practitioner Experiences Questionnaire (GPEQ)

**DOI:** 10.1186/1472-6963-10-108

**Published:** 2010-04-30

**Authors:** Oyvind A Bjertnaes, Arjan Nieland, Elisabeth Damerell, Andrew Garratt

**Affiliations:** 1 Department for Quality Measurement and Patient Safety, Norwegian Knowledge Centre for the Health Services, Oslo, Norway; 2 Innlandet Hospital Trust, Hamar Community Mental Health Centre, Hamar, Norway

## Abstract

**Background:**

Instruments have been developed to assess professional views of the quality of care but have rarely been tested for responsiveness to change. The objective of this study was to test the responsiveness of the General Practitioner Experiences Questionnaire (GPEQ) for the measurement of Community Mental Health Centres in Norway.

**Methods:**

National surveys were conducted in Norway in 2006 (n = 2,415) and 2008 (n = 2,209) to measure general practitioners' evaluation of community mental health centres. GPs evaluated the centres by means of a postal questionnaire, consisting of questions focused on centre quality and cooperation with GPs. As part of the national surveys 75 GPs in 2006 and 66 GPs in 2008 evaluated Hamar community mental health centre. Between the surveys, several quality improvement initiatives were implemented which were directed at cooperation with and guidance for GPs in Stange municipality, one of eight municipalities in Hamar centre catchment area. The main outcome measures were changes in GPEQ scores from 2006 to 2008 for GPs evaluating Hamar community mental health centre from Stange municipality, and changes in scores for GPs in the other seven municipalities and nationally which were assessed for statistical significance.

**Results:**

GPs in Stange municipality rated Hamar community mental health centre significantly better on the guidance scale in 2008 than in 2006; on a 0-100 scale where 100 represents the best possible experiences the score was 26.5 in 2006 and 58.3 in 2008 (p < 0.001). Apart from one item about workforce situation, none of the other scales and items showed significant changes. The control group from the other seven municipalities gave significantly poorer rating for the emergency situation scale, the workforce situation scale and seven items in 2008 than in 2006. The national results showed small differences between 2006 and 2008, even though several scales and items were significantly different. A question about changes in centre performance over the last 2-3 years showed that 82% of GPs from Stange municipality reported that Hamar community mental health centre had improved, compared to only 36% from the other seven municipalities and 40% nationally which was statistically significant.

**Conclusions:**

Following the implementation of an initiative designed to enhance service quality, the GPEQ identified expected changes in the guidance scale for the intervention group, indicating that the instrument is responsive to change. The worsening of services for GPs in the control group evaluating Hamar centre warrants further study.

## Background

Physicians and other healthcare professionals are frequently asked to participate in surveys to evaluate mental health care [1-7], but the quality of the measurement instruments in these studies is poorly documented. The lack of information relating to the measurement instrument including reliability and validity, raises serious questions about the credibility of findings. Several recommendations for the assessment of quality of measurement instruments exist [8,9], and core criteria are reliability, validity and responsiveness or sensitivity to change. Reliability is concerned with reproducibility and internal consistency, while validity concerns an instruments ability to measure what is intended [9]. There are several definitions of responsiveness but all relate to the ability of an instrument to detect clinically important change [8-10].

The General Practitioner Experiences Questionnaire (GPEQ) focuses on the assessment of Community Mental Health Centres in Norway on domains important for GPs satisfaction with the centres. The GPEQ has been tested for reliability and validity [11], but has not been tested for responsiveness to change. This is an important deficiency since the instrument should be appropriate for local and national projects evaluating changes over time including local assessment of the effectiveness of quality improvement initiatives. While several instruments that assess patient experiences and satisfaction with health care have been assessed for responsiveness [12,13], none of the instruments to assess physicians and other healthcare professionals' experiences and satisfaction with mental health care document this property [1-7,11].

As is the case for patient satisfaction surveys more generally, reports of general satisfaction have limited value in quality improvement processes [14,15]. Rather than simply asking GPs about their satisfaction with the community mental health centres, the GPEQ consists of concrete domains of care that are important as measures of service quality from the perspective of the GPs and hence contribute to their overall satisfaction with the centres. The GPEQ includes five scales that are supplemented with five individual items for use in repeated national surveys in Norway, as a result of the underlying development and validation work. The second national survey was conducted in 2008 [16].

The objective of this study was to test the responsiveness of the GPEQ for the assessment of Community Mental Health Centres in Norway. Between the national survey in 2006 and 2008, Hamar community mental health centre implemented several quality improvement initiatives in parts of the centre catchment area. Hamar community mental health centre has responsibility for general adult mental health services in eight municipalities in the South-East of Norway. The centre only had outpatients in the period 2006 to 2008, had no responsibility for emergency cases, and consisted of 41 employees in 2006. The new initiatives were directed at cooperation with and guidance for GPs in Stange municipality, one of eight municipalities in the centre catchment area.

The responsiveness of the GPEQ was assessed for GPs evaluating Hamar community mental health centre from Stange municipality in 2006 and 2008 who were included in a quality improvement initiative expected to improve the guidance scale and underlying items for the intervention group. GPEQ scores in 2006 and 2008 for GPs in Stange municipality evaluating Hamar centre were compared, in addition to comparisons between 2006 and 2008 for GPs in the control group consisting of GPs from the other seven municipalities in the catchment area, and comparisons over time at the national level.

## Methods

### Data collection

The data reported here were based on two national surveys in Norway in 2006 and 2008 among all regular GPs in Norway. A questionnaire that included the GPEQ was mailed to 3,704 GPs in 2006 and 3,942 GPs in 2008. Both surveys included a recommendation to take part in the survey by the leader of the Norwegian Association of General Practitioners. Non-respondents were sent three postal reminders in both surveys. The 2006 survey also included telephone reminders to postal non-respondents. The response rate in the national surveys was 65.2% in 2006 (n = 2,415) and 56.0% in 2008 (n = 2,209). An assessment of non-response bias in the 2006 material demonstrated adequate representativeness [17].

The procedure regarding informed consent, study design and data collection was approved by the Norwegian Social Science Data Services.

75 GPs in 2006 and 66 GPs in 2008 evaluated Hamar community mental health centre. The centre implemented several quality improvement initiatives in parts of the centre catchment area between the national survey in 2006 and 2008. The new initiatives were directed at cooperation with and guidance for GPs in Stange municipality, one of eight municipalities in the centre catchment area. They included half an hour daily telephone availability to the professional team at the centre for all GP's in Stange municipality and an offer of regular cooperation meetings each third month. The initiatives were not obligatory and GPs from two of five GP offices in Stange municipality participated in the cooperation meetings (7 of seventeen GPs). In addition the centre offered professional seminars for GPs in Stange municipality where GPs decided the topics, and these were conducted four times per year.

### General Practitioner Experiences Questionnaire (GPEQ)

The GPEQ [11] comprises the following scales which have good evidence for data quality, reliability and validity: workforce situation (4 items), discharge letter (3 items), competence (4 items), guidance (3 items) and emergency situations (2 items). Workforce situation includes items relating to stability in key positions and doctor coverage. Discharge letter includes items relating to quality, further plans and discharge letter time. Competence includes items relating to assessment and treatment skills. Guidance includes items relating to cooperation meetings, organised training and receiving necessary professional support. Emergency situations includes items relating to contact with and help from the centre in emergency situations. Scale scores are transformed to a 0 to 100 scale where 100 is the best possible rating. GP's with missing values on more than half of the items in a scale are excluded.

All scales met the criterion of 0.7 for Cronbach's alpha and test-retest correlations were 0.72-0.87. The results of construct validity testing were as hypothesised [11]. Five additional items including waiting time for patients are also part of the questionnaire. While scales are transformed to 0-100, item scores are shown in their original form as a five-point response scale 1-5. This is done to make it easier for the reader to distinguish between scales and items, and to adhere to common practice in this field. The 2008 survey also included a question about GP evaluation of the overall development of centre performance the last 2-3 years with the response categories "Much worse", "A little bit worse", "The same", "A little bit better", "Much better" [16].

### Statistical analysis

We constructed three groups: group 1, respondents from Stange municipality; group 2, respondents from the seven other municipalities in Hamar community mental health centres' catchment area; group 3, all responding GPs. We present means for all groups, both for scales and single items. For the intervention group and the two control groups we tested differences in scores for scales and items in 2006 and 2008 separately by means of t-tests. Differences between GPs in Stange municipality and GPs in the two other groups on the improvement question in 2008 were tested by means of the Pearson Chi-Square test. SPSS version 15.0 was used for statistical analyses.

## Results

GPs in Stange municipality evaluated Hamar community mental health centre significantly better in 2008 than in 2006 on the guidance scale (table 1). In 2006, the score for the guidance scale was 26.5 on a scale from 0 to 100 where 100 is best, compared to 58.3 in 2008 (p < 0.001). The other scales changed from 4 to ten points, but none were significant. Two of three guidance items and one item about workforce situation was better in 2008 than in 2006 (table 2).

**Table 1:** Scale scores for the two GP groups and nationally in 2006 and 2008

	Hamar centre, GPs in Stange municipality^a^		Hamar centre, GPS in other municipalities^a^		Nationally^a^	
	**2006 (n = 11)**	**2008 (n = 13)**	**2006(n = 64)**	**2008(n = 53)**	**2006(n = 2,415)**	**2008(n = 2,209)**

*Scale scores*:^b^						
Emergency situations	43.2	47.1	44.5	29.8**	52.2	50.8*
Workforce situation	39.2	49.0	51.1	39.1***	44.9	44.6
Discharge letter	64.4	57.7	57.3	55.3	52.2	53.4*
Competence	56.6	64.6	53.8	51.4	55.1	55.6
Guidance	26.5	58.3***	22.8	21.1	31.1	33.8***

^a^Tested differences between 2006 and 2008 for each group separately, independent samples t-tests. ***p < 0.001. **p < 0.01. *p < 0.05.

^b^Score 0-100 where 100 represent best possible experiences

**Table 2:** Item scores for the two GP groups and nationally in 2006 and 2008

	Hamar centre, GPs in Stange municipality^a^		Hamar centre, GPS in other municipalities^a^		Nationally^a^	
**Item scores:^b^**	**2006(n = 11)**	**2008(n = 13)**	**2006(n = 64)**	**2008(n = 53)**	**2006(n = 2,415)**	**2008(n = 2,209)**

						
*Emergency situations*:						
Ease of contact with the clinic in emergency situations	2.9	3.1	3.0	2.4**	3.2	3.1**
Help from the clinic in emergency situations	2.5	2.7	2.6	2.0**	3.0	3.0
						
*Workforce situation*:						
Do the clinic succeed in filling central professional positions	2.2	2.9**	3.0	2.5**	2.8	2.8
Good coverage of doctors at the clinic	2.2	2.7	2.7	2.2**	2.5	2.6
Stability in the professional executive positions at the clinic	2.9	3.1	3.3	2.8**	2.9	2.9
Stability in the professional positions at the clinic	2.9	3.2	3.2	2.7**	2.9	2.8*
						
*Discharge letter*:						
Quality of discharge letter	4.0	3.6	3.6	3.6	3.4	3.4
Plans for further follow-up in the discharge letter	3.7	3.3	3.2	3.1	3.0	3.1**
Receive discharge letter quickly from the clinic	3.0	3.0	3.0	2.9	2.8	2.9
						
*Competence*:						
Good competence to assess and treat patients at the clinic	3.5	3.9	3.5	3.4	3.4	3.4
Good professional advice from the clinic	3.1	3.7	3.0	3.0	3.1	3.2**
Patients got the necessary help when transferred from the clinic	3.3	3.0	3.1	3.0	3.2	3.2
Good cooperation between the professionals at the clinic	3.3	3.7	3.1	2.9	3.2	3.2
						
*Guidance*:						
Cooperation meetings with the clinic	1.7	3.4***	1.6	1.6	2.2	2.3**
Do the clinic offer organized guidance and professional seminars	1.7	3.3***	1.8	1.7	2.0	2.1***
Necessary professional support from the clinic	2.7	3.3	2.3	2.3	2.6	2.7**
						
*Other items*:						
General satisfaction	2.7	2.9	2.8	2.6	3.1	3.2*
Do the clinic reject patients you have referred	3.4	2.7	3.6	3.3	3.4	3.2***
Necessary feedback from the clinic during treatment	2.7	2.6	2.8	2.3*	2.7	2.4***
Contact with clinic in situations you need help	3.0	3.5	2.8	2.9	3.1	3.3***
Do the clinic take your opinions of the patients situation serious	3.5	3.4	3.3	3.4	3.4	3.4

^a^Tested differences between 2006 and 2008 for each group separately, independent samples t-tests. ***p < 0.001. **p < 0.01. *p < 0.05.

^b^Score 1-5 where 5 represent best possible experiences.

The guidance scale was almost unchanged from 2006 to 2008 for GPs in the control group evaluating Hamar centre; the guidance scale was 22.8 in 2006 and 21.1 in 2006 (table 1). However, two other scales were significantly poorer in 2008 than in 2006; the emergency situation scale fell from 44.5 in 2008 to 29.8 in 2008 (p < 0.01), while the workforce situation scale fell from 51.1 to 39.1 (p < 0.001). Seven items were also significantly poorer in 2008 than in 2006 for the control group (table 2), and six of these were related to the two poorer performing scales.

There were small differences between the national results in 2006 and 2008, but several scales and items were significantly different (table 1-2).

More than 80% of GPs in Stange municipality reported that Hamar Community Mental Health Centre had become much or a little bit better the last 2-3 years (table 3). In the control group for Hamar centre this only applied to around 36% and nationally the percentage was 40%, both being statistically different from the intervention group (p < 0.05).

**Table 3:** GPs evaluation of change in centre performance during the last 2-3 years for the two groups and nationally

	Hamar centre, GPs from Stange municipality % (n)	Hamar centre, GPs in other municipalities % (n)	Nationally
*Change*:^a^			
Much worse	0 (0)	6.4 (3)	5.6 (110)
A little bit worse	0 (0)	17.0 (8)	13.1 (258)
The same	18.2 (2)	40.4 (19)	41.5 (820)
A little bit better	54.5 (6)	31.9 (15)	31.4 (620)
Much better	27.3 (3)	4.3 (2)	8.5 (167)

^a ^The distribution for Hamar centre, GPs from Stange municipality, is significantly different than the distributions for the two other groups (p < 0.05, Chi-Square).

## Discussion

The purpose of the local quality improvement project was to identify improvement areas and develop initiatives to improve the poor scores for guidance and accessibility in the national survey in 2006. The quality initiatives that were implemented between the two national surveys were expected to result in improvements in the guidance scale for GPs from Stange municipality. We detected significant improvements on the guidance scale in the intervention group, thereby indicating that the instrument is responsive to change. This shows the ability of the questionnaire to measure changes that have actually happened [8]. As expected, the control group evaluating Hamar centre and the national results showed small differences on the guidance scale in 2006 and 2008.

The quality improvement project in Hamar community mental health centre applied a practical approach to quality improvement. The local quality initiatives were based on previous experiences including the national survey in 2006 and insights from cooperating with GPs. The initiatives had to be implemented without increased resources. The current study is not an evaluation of the effectiveness or efficiency of the local initiatives. These questions should be informed by literature reviews about cost-effective initiatives to improve collaboration between primary care and specialised mental health care identified within the scientific literature, including Cochrane reviews [18,19]. However, the large improvement in the guidance scale in the intervention group indicates the success of the local initiatives in relation to this aspect of the GPEQ. Therefore, centres aiming at improving GPs assessment of guidance from the centres could consider implementing the quality initiatives from Hamar community mental health centre. When the local project was finished the cooperation meetings was demanded and implemented at additional two GP offices, meaning that 15 of 17 GPs in Stange municipality participate in such meetings. A previous study has also confirmed the importance of guidance for GP satisfaction with Community Mental Health Centres [20].

The decline in scores for two scales in the control group was not expected in this study. However, employee data show that Hamar community mental health centre had a decline in the number of employees from 2006 to 2008 (18.2% decline). This might be an explanation for the significant decline in the workforce situation and emergency situations scale for the control group. The team working with the intervention group also had a decline in employees from 2006 to 2008 (12.5%), but did not experience significant changes in scores for these scales. One possible interpretation is that the local quality improvement project aimed at GPs from Stange municipality prevented the worsening of scores for workforce situation and emergency situations that can be expected as a result of the decline in employees. From January 2007 the team serving the intervention group also had more specialists per inhabitant than the rest of the centre, which also could be an explanation for the lack of decline in scores.

Poor scores for the GPEQ guidance scale in the 2006 survey led to the local quality improvement initiative on which this study was based. Our study showed a large improvement on the guidance scale in the intervention group, but also nationally the guidance scale showed largest improvement from 2006 and 2008. We currently lack information about quality improvement initiatives between the surveys from the other community mental health centres', but the national report from this project identified 11 centres with significantly different scores in 2006 and 2008 [16]. This inclusion of additional local quality improvement initiatives would have resulted in a more extensive evaluation of the GPEQ including the responsiveness of individual GPEQ scales that were targets for improvement. An electronic survey shall assess the importance and usefulness of the 2008 survey results to the community mental health centres. Data from this survey will inform further responsiveness testing of the GPEQ as part of the analysis of changes from 2008 to 2011 when the next survey is planned.

The sample in the intervention group was small. All scale scores for the intervention group were highest in 2008, but only the expected scale was significantly improved. The workforce situation scale improved as much as 10 points on the 0 to 100 scale from 2006 to 2008, and the small sample size might partly explain why this result was not significant. As mentioned we did not expect this scale to change, but further research is needed to assess potential positive unintended consequences of the local quality initiatives on other health care aspects. Further research should also be conducted to assess other aspects of responsiveness including the minimal important difference (MID), a concept related to estimating the smallest change in score that can be regarded as important [10]. In our study data protection issues made it impossible to connect responses on individual level between 2006 and 2008, consequently we did not have the opportunity of calculating responsiveness statistics.

The survey was undertaken over a two year period and the great majority of GPs are likely to have been present in both surveys. However, some score variation will be due to both the inclusion of new GPs and some GPs not responding to both surveys. These are potentially important considerations in the evaluation of responsiveness to change.

## Conclusions

Following the implementation of an initiative designed to enhance service quality, the GPEQ identified expected changes in the guidance scale for the intervention group, indicating that the instrument is responsive to change. The worsening of services for GPs in the control group evaluating Hamar centre warrants further study.

## Competing interests

The authors declare that they have no competing interests.

## Authors' contributions

OAB planned the paper together with ARN, ELD and AMG, carried out the statistical analysis, conducted the study together with administrative staff, and drafted the paper. ARN participated in the planning process, revised the draft critically and approved the final version. ELD participated in the planning process, revised the draft critically and approved the final version. AMG participated in the planning process, revised the draft critically and approved the final version. All authors read and approved the final manuscript.

## Pre-publication history

The pre-publication history for this paper can be accessed here:

http://www.biomedcentral.com/1472-6963/10/108/prepub
